# A guide to area‐restricted search: a foundational foraging behaviour

**DOI:** 10.1111/brv.12883

**Published:** 2022-07-12

**Authors:** Arik Dorfman, Thomas T. Hills, Inon Scharf

**Affiliations:** ^1^ School of Zoology, George S. Wise Faculty of Life Sciences Tel Aviv University 6997801 Tel Aviv Israel; ^2^ Department of Psychology University of Warwick Coventry CV4 7AL UK

**Keywords:** foraging mode, optimal foraging, intensive search mode, marginal value theorem, memory search

## Abstract

Area‐restricted search is the capacity to change search effort adaptively in response to resource encounters or expectations, from directional exploration (global, extensive search) to focused exploitation (local, intensive search). This search pattern is used by numerous organisms, from worms and insects to humans, to find various targets, such as food, mates, nests, and other resources. Area‐restricted search has been studied for at least 80 years by ecologists, and more recently in the neurological and psychological literature. In general, the conditions promoting this search pattern are: (1) clustered resources; (2) active search (e.g. not a sit‐and‐wait predator); (3) searcher memory for recent target encounters or expectations; and (4) searcher ignorance about the exact location of targets. Because area‐restricted search adapts to resource encounters, the search can be performed at multiple spatial scales. Models and experiments have demonstrated that area‐restricted search is superior to alternative search patterns that do not involve a memory of the exact location of the target, such as correlated random walks or Lévy walks/flights. Area‐restricted search is triggered by sensory cues whereas concentrated search in the absence of sensory cues is associated with other forms of foraging. Some neural underpinnings of area‐restricted search are probably shared across metazoans, suggesting a shared ancestry and a shared solution to a common ecological problem of finding clustered resources. Area‐restricted search is also apparent in other domains, such as memory and visual search in humans, which may indicate an exaptation from spatial search to other forms of search. Here, we review these various aspects of area‐restricted search, as well as how to identify it, and point to open questions.

## I. INTRODUCTION

**Fig. 1:**
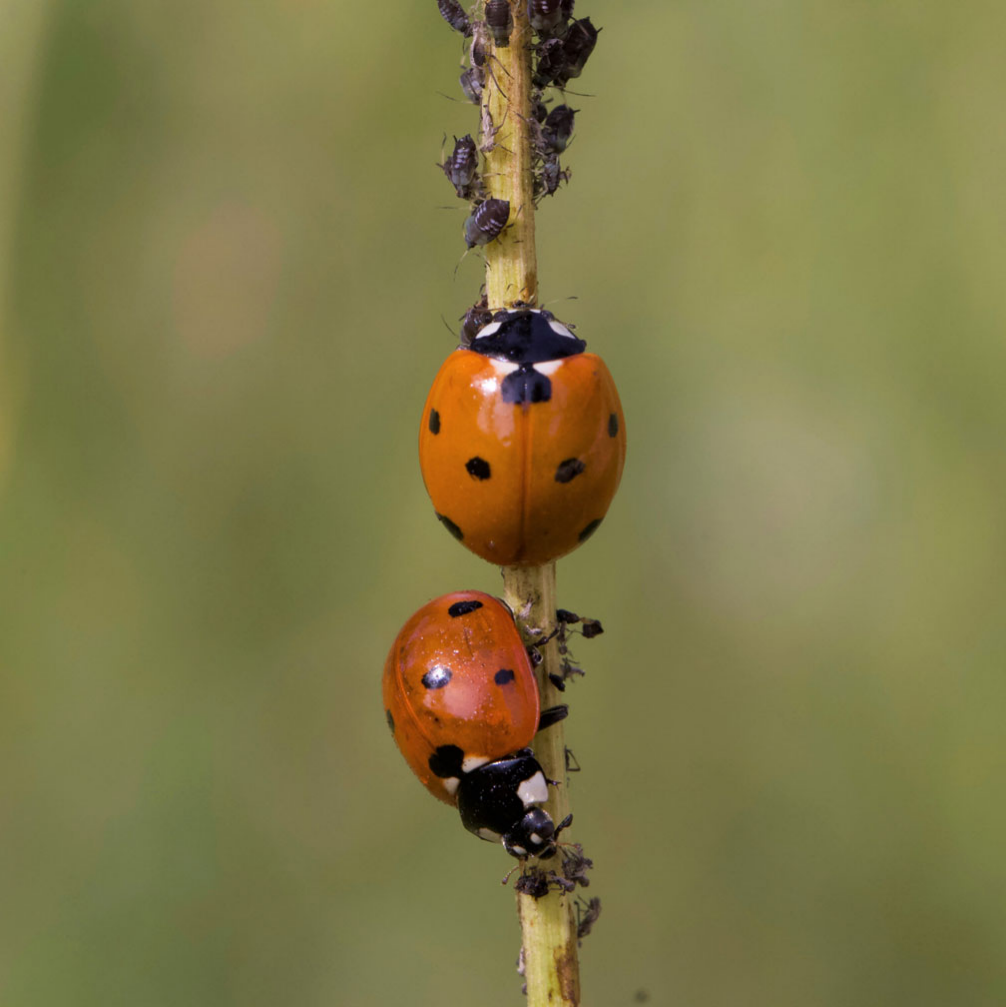
Two ladybird beetles (*Coccinella septempunctata*) searching for aphids on a stem. These beetles commonly use area‐restricted search (ARS) when foraging and have been intensively investigated in this context. Photograph, Bar Avidov.

Area‐restricted search (ARS) – also called area‐concentrated search, intensive search, or multi‐scale search (Tinbergen, Impekoven & Franck, 1967; Bond, 1980; Kölzsch *et al*., 2015) – is a common search pattern among motile organisms. It is reported in a vast variety of evolutionarily distinct taxa, including protists, nematodes, insects, birds, mammals, and humans (Visser, 1988; Nolet & Mooij, 2002; Hills, Brockie & Maricq, 2004; Pacheco‐Cobos *et al*., 2019). ARS is the process of switching back and forth from directional exploration (global, extensive search) to focused exploitation (local, intensive search) in response to resource encounters or associated expectations, which may be cued externally (e.g. by encounters with food) and internally [e.g. by memories/associations between food and external stimuli (Tinbergen *et al*., 1967; Schal *et al*., 1983)]. For animals searching in space for a resource, it involves the change from directional movement to a more tortuous movement, which enables the searcher to remain in a limited area, and then back to directional movement when resuming exploration. A common example of ARS is a ladybird beetle foraging for aphids (Fig. 1). In the absence of encountering aphids, it moves quickly in relatively straight paths, covering a greater distance (e.g. on a leaf), but after encountering aphids it makes many turns and reduces its movement speed, resulting in a thorough search in the area of the last find (Nakamuta, 1985; Ferran *et al*., 1994). After some period without encountering aphids, the ladybird resumes directional movement.

Area‐restricted search can also occur at multiple spatial scales. For example, many environments are composed of hierarchal patches, where the probabilities of encountering a cluster of resources are nested within one another (see Fig. 1 in Kotliar & Wiens, 1990). For a seabird looking for fish, a prey‐rich area of the sea, like a shelf edge or an upwelling, may be the first patch level. Within it, there are areas containing concentrations of fish schools, which comprise the second patch level, while each school is a patch of prey items (fish) – the third level. Many birds and other marine animals have been observed to feed efficiently in this habitat, intensifying search adaptively at each patch level (Weimerskirch, 2007; Paiva *et al*., 2010; Thums, Bradshaw & Hindell, 2011; Sommerfeld *et al*., 2013; Bailey *et al*., 2019).

Mathematical models, computer simulations, behavioural experiments, and observations of natural behaviour support the efficiency of ARS in habitats characterized by clumped resources (Bond, 1980; Haskell, 1997; Fauchald, 1999; Nolet & Mooij, 2002; Scharf, Kotler & Ovadia, 2009; Bailey *et al*., 2019). Such habitats are widespread among natural and manmade environments (Levin, 1994; Rietkerk *et al*., 2004; Wilke *et al*., 2018). Even when resources are not initially spatially clumped, foraging itself may create such patchiness (Motro & Shmida, 1995). Patch foraging models, such as the marginal value theorem (Charnov, 1976) can be adapted to incorporate ARS‐like behaviour for organisms unable to detect patch boundaries or when patch boundaries are fuzzy (Iwasa, Higashi & Yamamura, 1981; Adler & Kotar, 1999). ARS provides a spatially explicit mechanism for many optimal foraging strategies, which often do not explicitly state the mechanism by which an optimal giving‐up time can be achieved (Krebs, Ryan & Charnov, 1974; Ydenberg, 1984).

Considering the prevalence of ARS in nature as well as its utility across environments, including non‐spatial applications described below, there is a need to understand the biological properties of ARS better as well as how to identify it. Niko Tinbergen's four questions for studies of animal behaviour provide an outline (Tinbergen, 1963; Bateson & Laland, 2013): what is the adaptive value of the behaviour, does it change with ontogeny, how has it evolved, and what triggers and terminates it? In addition, we discuss tools for identifying ARS and open questions for future research.

## II. THE HISTORY AND PREVALENCE OF AREA‐RESTRICTED SEARCH

The first record of ARS is Laing (1937) concerning host‐finding patterns of the parasitoid wasp *Trichogramma evanescens*. Laing noticed that after contacting its host (eggs of the moth *Sitotroga cerealella*) the wasp reduced its movement speed and increased its turn rate, thus concentrating its search in the area of the last find. Other researchers later noticed the same pattern in other animals (Fleschner, 1950; Banks, 1957; Chandler, 1969; Murdie & Hassell, 1973; Smith & Sweatman, 1974). Tinbergen *et al*. (1967) coined the term ‘area‐restricted search’ in a paper addressing how animals adaptively space themselves to avoid predators using area‐restricted search. The term became popular in the ecological literature following Kareiva & Odell's (1987) modelling study of the ARS movements of predators. Bond (1980) introduced the terms ‘intensive’ and ‘extensive’ to refer to two stages of ARS (i.e. global and local search). Note that some researchers define only the intensive local search phase as area‐restricted search. We recommend against this use to avoid confusion with other forms of intensive search, such as random walks or periods of shortened path lengths in Lévy flights, and apply ARS herein to behaviours that adapt to local changes in resource density, including both extensive and intensive search.

ARS has now been reported in numerous animals, searching for a wide variety of targets in many habitats (Table 1). For example, in the ‘run and tumble’ behaviour of the bacteria *Escherichia coli*, individuals turn more frequently when moving down resource gradients than when moving up resource gradients (Macnab & Koshland, 1972). The nematode *Caenorhabditis elegans* uses ARS to find bacterial lawns on Petri dishes in the laboratory (and presumably to find resources in the wild; Hills *et al*., 2004). Global positioning system (GPS) studies on seabirds have recorded the use of ARS on multiple spatial scales, supporting the notion of hierarchal patch structure (Weimerskirch, 2007). Humans have also been observed to use ARS when foraging for resources both in virtual and real environments (Hills *et al*., 2013; Pacheco‐Cobos *et al*., 2019).

**Table 1:** Summary of key studies reporting area‐restricted search (ARS)

Resource type	Study systems	References	Comments
Prey	Active foragers (protists, worms, arthropods, gastropods, vertebrates)	Ferran *et al*. (1994); Hills *et al*. (2004); Nolet & Mooij (2002); Latty & Beekman (2009)	Common across all active foragers
Hosts	Parasitoids (wasps, flies)	Waage (1978); Strand & Vinson (1982); Tanaka *et al*. (2001); Chiu‐Alvarado *et al*. (2012)	Local search initiated mostly by odours
Nests	Central‐place foragers (ants, bees, isopods)	Hoffmann (1983*a*,*b*); Müller & Wehner (1988); Reynolds *et al*. (2007)	ARS used when a single resource item is searched for
Females	Males (insects; spiders)	Schal *et al*. (1983); Ono (1985); Rovner (1991); Hemptinne *et al*. (1996)	Local search initiated mostly by odours
Semantic memory	Humans	Hills (2006); Hills & Pachur (2012)	Memories are ‘searched’ using an ARS‐like strategy
*Factors triggering a local search*			
Prey capture	Active foragers (see *Resource type*, *Prey*)	see *Resource type*, *Prey*	see *Resource type*, *Prey*
Touch/contact with prey	Insects (beetles, flies)	Chandler (1969); Carter & Dixon (1984); Nakamuta (1985)	More than one cue or sensory mode may be involved
Odour/chemicals	Insects (wasps, true bugs, cockroaches)	Strand & Vinson (1982); Schal *et al*. (1983); Traczyk *et al*. (2020)	More than one cue or sensory mode may be involved
Memory/expectation/landmark	Active foragers (birds, fish), central place foragers (ants, bees)	Müller & Wehner (1994); Noda *et al*. (1994); Reynolds *et al*. (2007); Thums *et al*. (2011)	Memory of the location of either the nest or food source
Following other individuals	Active foragers (birds)	Einoder *et al*. (2011)	Other individuals initiating local search may indicate the presence of prey nearby
*Factors influencing ARS*			
Hunger	Active foragers (insects)	Bond (1980); Bell *et al*. (1985); Nakamuta (1985); Claver & Ambrose (2003)	Effects on local search initiation and its duration
Prey size/quality	Active foragers (protists, insects)	Glen (1975); Bell & Tortorici (1987); Latty & Beekman (2009)	Effects on local search initiation and its duration
Prey density/ clumpiness	Active foragers (insects, mites)	Eveleigh & Chant (1982); Tortorici & Bell (1988); Hemptinne *et al*. (1996)	Denser or more clumped prey leads to slower termination of local search
*Failure of ARS*			
Regular spatial pattern/isolated prey	Active foragers (fish, humans, ferrets, rodents)	Jenkins *et al*. (1995); Haskell (1997); Hill *et al*. (2002); Hills *et al*. (2013)	Moving away after encountering food was found to be a better tactic
Target easily detectable	Animals using vision (fruit bats, birds)	Tsoar *et al*. (2011); Flores‐Abreu *et al*. (2012)	Visible/observable target mitigates the need to use ARS

While ARS was originally described for spatial foraging, the behaviour associated with adaptively focusing a search around specific ‘areas’ in response to resource expectations has also been applied to non‐spatial searching. For example, ARS is used in problem‐solving (Smith, Huber & Vul, 2013), in which individuals search for a single word common to a set of three other words (e.g. what word is common to MOON, DEW, and COMB?). Humans also use ARS in memory search tasks, for example, when listing all the ‘animals’ they can think of, people tend to produce clusters of related items with transitions to new clusters as old clusters become depleted (Hills, Jones & Todd, 2012), a phenomenon first observed by Bousfield & Sedgewick (1944). ARS has also been observed in visual search, where prior expectations influence the movement of the eyes in a way that clusters saccades around previously found targets (Bailey *et al*., 2019). Similar ARS‐like behaviours can be found in optimal search algorithms in computer science. In simulated annealing, searching starts over a large area and then focuses on smaller more promising areas as the search continues (Kirkpatrick, Gelatt & Vecchi, 1983). A similar approach, termed ‘predatory search’, has been successfully applied to several optimization problems, such as the travelling salesman problem (Linhares, 1998; Liu & Wang, 2005).

## III. THE ADAPTIVE VALUE OF AREA‐RESTRICTED SEARCH

The widespread occurrence of ARS is hypothesized to be associated with its effectiveness in searching for clumped resources. Resources are often distributed in patches, with spatial autocorrelation between resource locations, and for such distributions strategies that localize foragers near resource clusters are likely to be most effective. According to optimal foraging theory, animals adapt their foraging behaviour to maximize resource intake (Stephens & Krebs, 1986; Pyke, 2019). A wide range of models have explored adaptive patterns of resource acquisition in patchy environments and many of these predict behaviour patterns consistent with ARS (see Table 2 for a summary of the predictions from selected models).

**Table 2:** Summary of predictions of selected models analysing area‐restricted search (ARS)

Predictions (system)	Study system	References
ARS should lead to predator aggregation in food patches	Ladybird beetles hunting aphids	Kareiva & Odell (1987)
ARS is efficient in patchy habitats. Its efficiency is higher when there are fewer patches, when each patch is larger, and when patch density is higher	General	Benhamou (1992)
ARS is more efficient when it is based on a memory of the target's general location than memoryless ARS when searching a novel landscape	Animals searching for a single hidden target	Benhamou (1994)
ARS works well for searching a random spatial pattern of resources because foragers create patchiness while using this search pattern, especially for long foraging bouts	Bees searching for rewarding flowers	Motro & Shmida (1995)
The tortuosity of a local search should decrease and step length should increase when prey become less clumped	Ferrets searching for hidden prey	Haskell (1997)
ARS is most efficient for intermediate levels of prey aggregation	Marine predators of krill and schooling fish	Fauchald (1999)
ARS is efficient when prey is clumped, both in terms of net gain and gain per movement	Copepods searching for food	Leising (2000)
When food is detected, ARS is more efficient than other search patterns, such as continuously directional or tortuous movement, assuming that food is clumped in space	Swans searching for tubers buried in lake sediment	Nolet & Mooij (2002)
ARS following prey encounter is more efficient than a constant directional or tortuous movement. ARS based on information on other prey items nearby is more efficient than switching to local search per encounter	Flatfish searching for bivalves or worms on the sea bottom	Hill *et al*. (2003)
When resources are distributed in a regular spatial pattern, a ‘lose–stay, win–shift’ strategy should be favoured over ARS	General	Scharf *et al*. (2009)
ARS benefits increase with patch clumpiness; a gradual transition from local search to directional movement is better than an abrupt switch; when patch density increases, a brief local search is preferred over longer ones	General	Bartoń & Hovestadt (2013)
ARS is efficient when prey is clumped. Switching between local and extensive search and *vice versa* based on non‐directional sensory cues (e.g. smell) is more efficient than switching based on an encounter with food and giving‐up time	General	Nolting *et al*. (2015)
For mesopredators, ARS should be under less strong selection if top predators learn to find mesopredators inside their prey patches	General	Scharf (2021)

The marginal value theorem models optimal patch departure time based on rate of resource acquisition within patches and travel time between patches. To maximize their rate of resource intake, foragers should stay within a depleting patch until the rate within that patch matches the average rate of resource intake across all patches, including travel times between patches (Charnov, 1976). For animals unable to detect patch boundaries, ARS is an effective way to approximate the patterns predicted by the marginal value theorem. For example, a model by Adler & Kotar (1999) demonstrated that animals unable to detect patch boundaries could nonetheless act approximately as predicted by the marginal value theorem by controlling the rate at which they ‘get lost’ during intensive search (i.e. wander beyond the patch boundary) before moving more directionally to another patch.

Computer simulations and models have validated the efficiency of ARS (Benhamou, 1992; Haskell, 1997; Fauchald, 1999; Leising, 2000; Nolet & Mooij, 2002; Scharf *et al*., 2009). For example, Scharf *et al*. (2009) [see also Hills (2006)] used a simulated genetic algorithm to generate optimally foraging ‘organisms’. The simulated environment consisted of a grid containing differently distributed ‘prey’ items. The forager's movement was controlled by three parameters: (1) direction of movement before an encounter; (2) direction after an encounter; and (3) duration of movement after an encounter. Foragers evolved to have more tortuous movement paths after prey contact than before prey contact when the prey distribution was clumped but moved in a more ballistic fashion before and after encountering prey when the prey was abundant and randomly distributed. Bartoń & Hovestadt (2013) found similar results but treated the transition from local search to global search as gradual rather than abrupt, and dependent on the forager's internal state rather than only on prey detection [see also Kareiva & Odell (1987)].

Fauchald (1999) showed that ARS is efficient in a simulated environment constructed as a hierarchical patch system. ARS occurred naturally at several scales when efficient search was of ‘adaptive value’ in the simulation. Leising (2000) constructed a model aiming to measure the efficiency of ARS in copepods foraging for patchily distributed phytoplankton. The model consisted of a square two‐dimensional grid, with programmable food concentrations and distributions, and a copepod movement algorithm. The model explored two distributions of prey items (patchy *versus* evenly distributed) with copepods either using ARS (increasing their turning angle and decreasing their step length after encountering prey), or not. The results showed that copepods foraging using ARS in a patchy environment had the highest foraging efficiency. This model is likely to be applicable to other examples of foraging in a 2D environment.

An alternative to ARS is the Lévy walk (Humphries & Sims, 2014; Kölzsch *et al*., 2015). Lévy walks are random walks in a direction chosen at random and with path lengths taken from a power‐law density distribution (a Lévy distribution) $P\left(l\right)=al^{-\mu }$, where *l* is the path length of each step, *a* is a normalization constant, and *μ* is a scaling parameter. In the biological realm, *μ* lies between 1 and 3 (Pyke, 2015; Zaburdaev, Denisov & Klafter, 2015). Many studies have found that foraging animals move in a Lévy‐like manner with *μ* ≈ 2 in patchy environments (Reynolds, Schultheiss & Cheng, 2014; Kölzsch *et al*., 2015). A Lévy walk results in episodes of large‐scale directional movements, punctuated by episodes of small‐scale tortuous movements – similar to ARS. For this reason, ARS and Lévy walk are sometimes used interchangeably. However, this is misleading as Lévy walks (or Lévy flights) have no memory component: new directions and path lengths are chosen without reference to resource encounters or expectations. After a long path without encountering resources, the probability that an organism using a Lévy walk will engage in an intensive (short path length) search remains the same. An animal using ARS, however, should only initiate a local search if environmental cues predict resource availability nearby.

ARS can produce movement patterns with power‐law‐distributed path lengths that superficially resemble Lévy flights (Hills *et al*., 2013). If researchers only consider the movement patterns (path length distribution), ARS and Lévy flights can look identical. Thus, path length distribution is not sufficient to separate ARS from Lévy walks. Instead, researchers must monitor resource distributions and examine how animals modulate path length and turning angle in relation to recent encounters with resources. Many researchers have questioned the validity of Lévy‐walk models in describing animal foraging (Benhamou, 2007; Codling, Plank & Benhamou, 2008; Pyke, 2015; but see Reynolds, Leprêtre & Bohan, 2013). Moreover, direct comparisons between Lévy foraging and ARS find a distinct performance advantage for ARS when resources are clustered (Plank & James, 2008; Ferreira *et al*., 2012).

Various experiments have supported the predictions of ARS models and the efficiency of ARS. For example, Haskell (1997) tracked ferrets (*Mustela putorius furo*) in arenas with different distributions of ‘prey’ items (oil droplets). The ferrets' search became more tortuous as the ‘prey’ became more aggregated, as predicted by models (e.g. Leising, 2000; Scharf *et al*., 2009). In another study, Bewick's swans (*Cygnus columbianus bewickii*) searching for tubers buried in lake sediments used ARS that corresponded with the size of the tuber patches (Nolet & Mooij, 2002). A computer simulation confirmed that this search pattern was more efficient than the continuous use of tortuous movement without intervening extensive search periods. The hunger level of green lacewings (*Chrysoperla carnea*) and assassin bugs (*Rhynocoris marginatus*) was found to affect the duration, turning rate, and area of the intensive search periods (Bond, 1980; Claver & Ambrose, 2003). Food deprivation resulted in increased speed during global search and longer periods of local search after prey contact. The authors concluded that this foraging behaviour pattern was the best fit to a poor environment characterized by small and sparse food patches. Thus, the lacewings tailored ARS length and intensity to fit their expectations of habitat structure. Furthermore, as some animals switch to local search following an encounter with specific prey items but not with others, it has been suggested that they may possess prey‐specific spatial pattern expectations and switch to local search only if the prey items are expected to be clumped (Déjean, Lachaud & Beugnon, 1993; Eifler *et al*., 2012).

In an experiment on humans ‘foraging’ for invisible recourses in a virtual environment (Hills *et al*., 2013), when the resources were clumped humans used ARS within patches. However, when the resources were randomly distributed, the search was solely global/extensive, with significantly fewer turns, and local search was not used. Ross & Winterhalder (2018) followed two Colombian blowgun hunters while carrying GPS loggers and recording prey contacts. They found that hunters reduce their speed and increase their turning angle as a function of prey encounters, resulting in a thorough local search in resource patches, such as fruiting trees, where their prey is often found. In a study examining the foraging behaviour of the copepod *Acartia clausi*, it was found that copepods placed in a vessel without any food (phytoplankton) do not use ARS, while copepods placed in vessels containing various concentrations of phytoplankton use ARS and stay in a food patch to satiation (Leising & Franks, 2002). Furthermore, the ARS was more prominent (turning angles increased and speed decreased) when they were starved for a longer time.

## IV. DOES AREA‐RESTRICTED SEARCH CHANGE WITH ONTOGENY?

Mendez, Prudor & Weimerskirch (2017, 2020) provide the most detailed example of ontogenetic change in ARS: red‐footed booby (*Sula sula*) juveniles spend longer in local search than adults. The reason for using more tortuous movement could be a reluctance or inability to depart too far from the colony or their inexperience as foragers. This difference may perhaps reduce competition for food between age classes within the colony. Grey seal (*Halichoerus grypus*) pups post weaning change from mostly uniform movement (constant movement speed and tortuosity) to an ARS strategy: global search (fast, directional movement) and local search (slow, tortuous movement; Carter *et al*., 2020). Wandering albatrosses (*Diomedea exulans*) of all ages engage in ARS, but younger birds less than 16 years old use local search over larger areas than older ones, suggesting they may learn how prey are distributed over time or adapt their search to other age‐related physiological properties (Weimerskirch *et al*., 2007). Very young albatrosses (i.e. during the first months of independence) do not use ARS at all and do not engage in local search (Riotte‐Lambert & Weimerskirch, 2013).

Life history studies often reveal patterns of more exploration early in life followed by more exploitation later. Foragers that explore more during early life acquire more information about global resource levels and tend to benefit from this information later in life with increased resource intake (Eliassen *et al*., 2007). This pattern may be mirrored in information foraging more broadly. For example, studies of human children reveal distinct patterns of persistent exploration during problem solving that become more exploitative as individuals age (Gopnik *et al*., 2017; Gopnik, 2020).

Studying ARS throughout ontogeny remains a research area with numerous opportunities and an important future direction towards understanding its innate and learned components. In parallel, studying naïve individuals during their first foraging bouts may indicate whether ARS is an innate behaviour. For example, ARS in honeybees is probably innate: naïve bees used ARS even when flowers were distributed randomly (Keasar, Shmida & Motro, 1996).

## V. HOW HAS AREA‐RESTRICTED SEARCH EVOLVED?

In order for ARS to evolve, certain conditions must be met: active search, absence of accurate sensory cues, an expectation to find a resource and, when searching for multiple targets, a clumped or clustered resource distribution.

Almost all records of foraging‐related ARS concern active foragers, irrespective of whether they search for multiple targets or one. However, there may be exceptions. For example, Bennison *et al*. (2019) tracked Atlantic puffins (*Fratercula arctica*) using GPS loggers and time‐dive recorders. After leaving the colony at the start of a foraging trip, puffins often land on the water and allow tidal currents to drift them passively towards prey patches. Thus, while they are not ‘actively’ foraging, the currents allow them to carry out a local search.

Another prerequisite for the evolution of ARS is an inability to sense the target directly. If the target location is known, or there is a reliable cue leading directly to the target, search based on taxis or beckoning is likely to be more efficient (e.g. Grünbaum, 1998). All examples of ARS involve targets with short detection ranges compared to the distance between resource items. For example, the prey may be hidden and can be found only by touch when it is buried under sand, snow, or water (Fortin, 2002; Hill *et al*., 2002). Examples of ARS use in these circumstances include gophers (*Geomys bursarius*) foraging in underground burrows for roots, and Bewick's swans foraging for tubers buried in lake sediments (Benedix Jr, 1993; Nolet & Mooij, 2002). In marine habitats, the prey‐detection range is often limited either by the seawater medium compromising visibility, or because prey density varies greatly (Sims *et al*., 2008). Thus, when the predator cannot immediately locate the next prey item following the first encounter, shifting to local search may be optimal.

Animals engage in local search when there is a greater probability of encountering a resource (Hills, 2006). Animals often use ARS while foraging in a patchy habitat (Lode, 2000; Weimerskirch *et al*., 2007; Paiva *et al*., 2010; Regular, Hedd & Montevecchi, 2013; Pacheco‐Cobos *et al*., 2019). Where there is spatial autocorrelation of prey (i.e. in patchy habitats), the location of a single prey item triggers an expectation to find additional prey nearby. Although patchy habitats are considered ubiquitous in nature and foraging models assume patchiness (Charnov, 1976; Benhamou, 1992; Levin, 1994), there are exceptions, such as the regular spatial pattern of desert shrubs competing for water (Beals, 1968). In a regular spatial pattern – where a resource encounter predicts the *absence* of other resources nearby – animals are expected to use the opposite behaviour to local search following an encounter with food, i.e. they should move away (Krakauer & Rodríguez‐Gironés, 1995; Scharf *et al*., 2009).

ARS is also likely to evolve in non‐patchy environments when the search is for a single target that is difficult to locate. For example, navigating *Cataglyphis* desert ants use a diverse toolkit including path integration, a celestial compass, and an odometer to navigate back to their nest along a shorter, often straight, path after finding food (reviewed in Wehner, 2003). When an ant reaches its predicted destination but does not encounter the nest entrance (e.g. due to small errors), it moves in spirals until it locates the entrance (Müller & Wehner, 1988). A similar search pattern was reported for isopods (*Hemilepistus reaumuri*) returning to their nest (Hoffmann, 1983*a*,*b*) and honeybees (*Apis mellifera*) when searching for food at a known location (Reynolds *et al*., 2007). The predicted target location here presumably involves an imprecise location memory (Hoffmann, 1983*a*,*b*). Another example of ARS involving a single target is a male spider courting a female; males use a movement pattern similar to local search to remain in the latter's vicinity (Rovner, 1991). There are several differences between the type of ARS used to search for a single target compared with multiple targets. When searching for multiple resources, the trigger for a local search is often an encounter with the first resource whereas searching for a single target requires use of other cues that indicate it is nearby, and that local search should commence. Searching for multiple targets often involves a longer sequence of local search episodes interspersed with more directional movement, whereas searching for a single target involves a shorter sequence and ends with target discovery.

## VI. WHAT TRIGGERS AND TERMINATES AREA‐RESTRICTED SEARCH?

When animals search for hidden targets, how do they know they are getting closer? In other words, what are the triggers to switch from an extensive, global search to an intensive, local search? The answer relies on a combination of sensory cues and memory.

Sensory cues, such as smell, touch, or vision, may indicate (e.g. through associative memory) the presence of a nearby resource and initiate local search. In ladybird beetles (*C. septempunctata*) using ARS, for example, the touch or taste of prey trigger local search (Nakamuta, 1985). Contact of the forager with a prey item, even without consumption, can elicit local search (Chandler, 1969; Carter & Dixon, 1984), although feeding on the prey results in a longer episode of local search in ladybirds (Nakamuta, 1985). A larger or more nutritious prey item was found to result in a longer local search in the lizard *Pedioplanis namaquensis* feeding on termites *versus* rice, the true bug *Blepharidopterus angulatus* feeding on fourth‐instar *versus* first‐instar aphids, and the fruit fly *Drosophila melanogaster* feeding on a higher or lower concentration of sugar water (Glen, 1975; Bell & Tortorici, 1987; Eifler *et al*., 2012). Male insects, such as German cockroaches (*Blattella germanica*) or two‐spot ladybird beetles (*Adalia bipunctata*), use ARS to find mates (Schal *et al*., 1983; Hemptinne *et al*., 1996). Here, the trigger for local search is the female sex pheromone, which is non‐volatile so preventing the use of chemotaxis as a more direct search strategy. Interestingly, removing the female after pair formation in two termite species leads to ARS behaviour by the male but removing the male does not have the same effect on the female (Mizumoto & Dobata, 2019). Similarly, parasitoids respond to host chemical cues, such as gland secretions, by moving more slowly, turning more frequently, and inspecting the surface with the ovipositor [see Waage (1978) and Strand & Vinson (1982) and references therein].

It is not always clear whether a switch to local search is triggered by sensory cues or what these cues are. For example, mud snails (*Hydrobia ulvae*) switch to tortuous movements and local search when encountering food but also sometimes in the absence of such an encounter (Kölzsch *et al*., 2015). This may indicate that the switch to local search may be not only reactive but also generated by internal cues (Kölzsch *et al*., 2015). Perhaps the sensory cues responsible for this switch remain to be identified, as they are difficult to rule out. Another hypothesis is that this switch is an example of a ‘vacuum behaviour’ (Lorenz, 1981). According to this hypothesis, in the absence of a releasing stimulus for local search in the experimental arena, the snails lower their thresholds for engaging in a local search, eventually performing it spontaneously, without any stimulus. This may be a difference between ARS and Lévy walks: ARS requires some external cue to initiate local search whereas Lévy walks do not. An animal engaging in alternating exploration and exploitation in the absence of sensory cues is consistent with a Lévy walk.

Memory may also play a more explicit role in ARS. Foragers that exhibit site fidelity engage in local search when they arrive at a memorized feeding site (Zach & Falls, 1976; Noda *et al*., 1994; Thums *et al*., 2011). Common guillemots (*Uria aalge*) tracked using GPS loggers and temperature‐depth recorders (Thums *et al*., 2011) did not use ARS at large or medium scales, for which they used a commuting motion flying in a straight line to the same feeding grounds repeatedly. Only upon arrival to within a ~2 km radius of their regular feeding grounds did the birds switch to local search. Thus, it appears that these birds can remember the approximate location of the feeding grounds and use a local search to find prey only when they know they are nearby. A similar foraging pattern was found in southern elephant seals (*Mirounga leonina*) which swim directly to a preferred location, south of Antarctic Circumpolar Current fronts, where they switch to intensive searching (Regular *et al*., 2013). However, in contrast to the guillemots, these seals also feed on their way to and from the feeding grounds.

Ants and bees use ARS when they fail to locate a target at the end of a memorized track (Müller & Wehner, 1994; Reynolds *et al*., 2007). Navigating desert ants (*Cataglyphis* or *Melophorus* spp.) spiral around their estimated nest entrance position at the end of their path integration route, and honeybees use a similar search pattern when re‐locating a known food source. Reynolds *et al*. (2007) trained honeybees to consume honey from a feeder near the hive. The bees remembered its location and flew in straight paths towards it. When the feeder was removed, the bees performed a local search in the former area of the feeder for some time before giving up and returning to the hive.

Termination of local search is important to avoid spending too long in a food patch that has been exhausted and is no longer profitable. Termination will depend on both intrinsic and extrinsic factors. A common example of an intrinsic factor is hunger level: hungry animals persist for longer in local search than satiated ones (Bond, 1980; Carter & Dixon, 1982; Bell *et al*., 1985). Regarding extrinsic factors, local search duration can be correlated with prey quality, density, and level of clumpiness; the likelihood of its termination increases with time since last encounter (Eveleigh & Chant, 1982; Nakamuta, 1985; Bell & Tortorici, 1987; Tortorici & Bell, 1988). Some foraging models predict that animals use the time since last encounter as a patch‐departure rule (Krebs *et al*., 1974; Ydenberg, 1984), which may be expressed as a switch from local to global search. Another potential mechanism is to use the time since last encounter in parallel with an habituation process: an encounter with prey upon arrival at a patch would lead to longer residence time than an encounter later on (Bell, 1990). In tropical ants (*Paraponera clavata*), local search lasts longer following a single food reward than after several successive rewards (Breed *et al*., 1996). The neural mechanism of local search termination has been investigated in *C. elegans*: local search is terminated by release of a RFamide neuropeptide (flp‐18) from specific neurons (AIY neurons) (Cohen *et al*., 2009). In *D. melanogaster*, genetic differences are involved: larval ‘sitters’ remain longer in local search than ‘rovers’ (Bell, 1985). Finally, Visser (1988) suggested that the shift from local search to global search is characterized by a shift from reliance on ‘idiothetic circling’ to ‘allothetic control’ (i.e. a shift from internal signals to external cues).

## VII. A NEURAL MODEL FOR AREA‐RESTRICTED SEARCH

ARS is used across the kingdom of life, even by protists (Latty & Beekman, 2009), and therefore does not require a central nervous system. However, among metazoans, there do appear to be common neural pathways controlling ARS. Hills *et al*. (2004) performed an in‐depth exploration of the neural architecture of ARS in the nematode *C. elegans*. Wild‐type worms employ local search when encountering prey (*E. coli*). However, mutant *C. elegans* with genetically impaired glutamate sensing and transport, wild‐type worms with ablated dopaminergic neurons, and worms that were incubated with a dopamine antagonist, were unable to switch to local search. Moreover, incubation in dopamine restored the ablated worms' wild‐type phenotype but not the phenotype of the glutamatergic mutants, suggesting that glutamatergic neurons and dopaminergic neurons operate in the same pathway. The researchers proposed a model according to which dopaminergic sensory neurons, which respond to prey capture, upregulate glutamatergic interneurons. These in turn activate motor neurons that control turning.

A dopaminergic pathway may underlie a general neural architecture controlling ARS‐like behaviour across a wide variety of species, ranging from nematodes to mammals (e.g. Anderson, Braestrup & Randrup, 1975; Szczypka *et al*., 1999; Due, Jing & Klaudiusz, 2004; Hills *et al*., 2004; Hills, 2006). Similar dopaminergic pathways also modulate addiction (Berke & Hyman, 2000), and this too may be related to ARS. For example, cocaine application to wild‐type *D. melanogaster* increases turning frequency (Bainton *et al*., 2000). Inhibiting dopamine synthesis prevents this response, but treatment with L‐dopa restores it. The dopaminergic modulation of reward‐related sensitivity is a common feature across metazoans and may be related to general goal‐directed control of attention in humans as well as associated pathologies [reviewed in Hills (2006), Winstanley *et al*. (2012) and Hills, Todd & Jones (2015)]. For example, in obsessive–compulsive disorder (OCD) local search is too long, and in attention deficit hyperactivity disorder (ADHD) it is too short. Both conditions also show a predictable role for dopamine based on its modulation of ARS, as do other human goal‐directed pathologies (Hills, 2006). Hills (2006) suggests this supports a shared evolutionary mechanism across species – dopaminergic modulation between exploration and exploitation – and the evolution of goal‐directed attention. However, any involvement of the hippocampus and other neural regions dedicated to internal representations of information (such as space, time, and value; Landi & Buffalo, 2022) has yet to be explored with relation to ARS.

## VIII. CLASSIFYING AREA‐RESTRICTED SEARCH

How can researchers identify ARS? One key aspect is confirmation that an animal responds to a potential resource by intensifying search nearby. This can involve an increase in turning angle or a reduction in speed following a resource encounter. For example, Hills *et al*. (2013) distinguished ARS from Lévy walks by measuring turning angle immediately after resource encounters. In clumped resource distributions, turning angles were higher following a resource encounter, but in more regular resource distributions, there was no change in turning angle before and after a resource encounter.

Several excellent reviews of ARS detection methods are available (Knell & Codling, 2012; Edelhoff, Signer & Balkenhol, 2016; Bennison *et al*., 2018), so we restrict ourselves here to a general discussion. Movement data, whether GPS (Weimerskirch, 2007), video (Kölzsch *et al*., 2015), manual (Bond, 1980), or from animal tracks (Subach, Scharf & Ovadia, 2009) consist of the coordinates of the animal with a timestamp for each coordinate. From such data, a path can be easily constructed, and can then be described using parameters such as turn angle and speed, depending on the measurement scale [see Edelhoff *et al*. (2016) for a detailed list]. These can be sorted into different categories or behavioural states based on statistical differences between segments. The segmentation and the exact parameters used are specific to each method. For example, Dzialak *et al*. (2015) analysed the foraging paths of greater sage‐grouse (*Centrocercus urophasianus*) fitted with GPS recorders that recorded their location every hour. Then, for every set of coordinates, they calculated the relative displacement between sequential GPS coordinates as parameters for segmentation into within‐patch (local search) and between‐patch search episodes. From a visual assessment of the data, the researchers determined that a relative displacement of less than 5% represented local search. This probably holds true in this case because the birds were not travelling at constant speed between patches, as opposed to within patches. This method, however, is probably overfitted to the described study and may not capture ARS patterns in other species.

More generally, methods for detecting ARS must be adapted to the study animal and the scale of their foraging in the relevant habitat (Knell & Codling, 2012). For example, first passage time (FPT) is the time taken for an animal to cross a circle of a given radius from its starting location (Fauchald & Tveraa, 2003). When performing a local search, FPT will be significantly larger than when performing a global search. However, the circle radius chosen must be appropriate to the scale of the animal's ARS, and multiple radii may be required to identify it. It is also necessary to use equipment with an appropriate spatial resolution and sampling rate. For example, for GPS tracking data, FPT is less effective at detecting small‐scale ARS, as the effect of GPS spatial error and low sampling rates increases as scale decreases (Pinaud, 2008). In addition, GPS logger accuracy depends on factors such as the animal's proximity to the sea surface and weather conditions, requiring suitable validation. In a GPS study on the masked booby (*Sula dactylatra*) researchers used FPT to classify ARS segments, while simultaneously using data from time‐depth recorders to distinguish between diving (feeding) events and periods during which birds were resting on the water surface (Sommerfeld *et al*., 2013). More than 70% of segments classified as local search by FPT were, in fact, resting periods without any feeding dives. This study demonstrates the need for validation when classifying behaviour using location data. Finally, accelerometry, which measures changes in movement velocity (and sometimes also movement direction), has become a popular tool in studies of movement ecology (Brown *et al*., 2013; Leos‐Barajas *et al*., 2017). This tool may prove to be useful in studying ARS, and specifically transitions between local search and directional, faster movement after leaving a patch.

## IX. DISCUSSION

ARS is a movement pattern found in numerous and diverse animal taxa. It is characterized by concentrating search effort in a restricted space in direct response to resource encounters or cues. This review focuses on providing an overview of the prevalence, adaptive value, mechanisms, and methods for detecting ARS. We also note that the shared mechanisms underlying exploration and exploitation across metazoans imply a long evolutionary history for ARS. Similar descriptions and underlying neural architectures for attention and goal‐directed behaviour suggest that ARS may have been exapted for controlling search and attention in non‐traditional foraging environments, such as in the control of visual attention and internal ‘mental’ search (Hills & Butterfill, 2015). For example, the ‘win–stay lose–shift’ model of decision making, whereby people adhere to choices until they fail to be rewarding, as well as reinforcement learning, which leads to repetition of behaviours that have been successful in the past, are potentially related to ARS, both in terms of their adaptive value and the underlying neurophysiology (Worthy & Maddox, 2014). ARS likely has a rich evolutionary history, giving rise to putative exaptations, establishing it as a core foraging behaviour – potentially the default foraging behaviour from which others have diverged.

ARS is adaptive because it optimizes a search for nearby targets when there is limited information on their location: an animal looking for prey in a patchy habitat should expect to encounter further prey near those already discovered, and an animal looking for a single resource in a known area (e.g. at the end of path integration) should also expect to find its target near its approximate location. Non‐ARS behaviours are typically observed in cases where animals do not engage in active search, resources are not clustered, or when animals know exactly where resources are located. In the latter cases, it would be more efficient to use taxis instead (Grünbaum, 1998). Furthermore, ARS should fail when the spatial distribution pattern of prey is regular (Jenkins *et al*., 1995; Scharf *et al*., 2009).

The mechanism triggering local search should be a reliable cue of a nearby target. Some animals rely on sensory cues, such as touch, taste, or smell of food, while others rely on intrinsic cues like a memory of the target site (Nakamuta, 1985; Reynolds *et al*., 2007). The neurological mechanism underlying this behaviour involve dopaminergic and glutaminergic neurons, which are believed to be related to the reward anticipation pathway (Hills, 2006).

This review is framed in terms of Tinbergen's four questions: we address the mechanisms that underly the behaviour, its adaptive value, some aspects of its evolution, and how it changes during ontogeny. There remain many questions for future study. For example, to what extent are ARS‐related behaviours homologous across species, driven by evolution from a common evolutionary precursor, or convergent, involving independently evolved biological mechanisms adapted to a similar function? To what extent are ARS and Lévy walks related: could Lévy walks be the physiological default state of an optimally sensitive ARS (e.g. Abe, 2020)? In addition, since ARS can be mistaken for Lévy walks when researchers fail to assess resource distributions, how many records of Lévy walks in the literature actually represent observations of ARS (Hills *et al*., 2013) or animals responding to clumped resource distributions?

While Hills (2006) provided evidence for shared ARS‐like behaviour and underlying neural architectures across species, much is left to understand. For example, studies of experimental evolution when food is provided in a clumped or regular pattern could help to uncover the heritability and selective forces for ARS. How ontogeny affects ARS is also poorly understood: do organisms in different environments tune ARS optimally over their life history? For example, if hunger levels change with ontogeny, how does this affect ARS?

It would be intriguing to examine the behavioural repeatability of ARS. For instance, do some individuals use ARS more than others? If so, we should be able to correlate a tendency to initiate ARS with other foraging characteristics. Another potentially fruitful direction will be to apply cost–benefit analyses to ARS. While its benefits under specific circumstances are clear, movement costs have not yet been thoroughly integrated into models and may affect the tendency to engage in local search and its duration. For example, frequent turning, which characterizes local search, increases movement costs (Halsey, 2016). Furthermore, predation or other risks could be higher during local foraging *versus* more directed movement: the forager's own predators could locate it within food patches and this might select against the use of ARS (Sih, 2005; Scharf, 2021). Alternatively, movement in open areas outside food patches could be more dangerous (Kotler, Brown & Hasson, 1991).

There are a number of additional factors likely to influence ARS, such as learning and the presence of conspecifics. The idea that ARS improves with learning is not new (e.g. Haskell, 1997), but examining how fast animals can learn to use ARS in suitable habitats is of interest to understand how flexible their foraging behaviour is and to separate between innate and learned behaviour. Other neglected aspects, such as the rate of ARS forgetting after learning, how rates of environmental change affect both the learning of ARS and its forgetting, and whether animals rely on landmarks or use other navigation methods before switching to local search.

This review also focuses on solitarily foraging individuals. There are some reports of animals switching to local search while following other individuals that have already switched (Einoder *et al*., 2011). Indeed, many animals tend to join conspecifics when they encounter food (Giraldeau & Beauchamp, 1999; Prokopy & Roitberg, 2001). Thus, there may be an argument for merging ARS with the producer–scrounger phenomenon, in which some animals search for food while others follow and join them if food is found (Barnard & Sibly, 1981). Initiating local search when observing others *versus* not doing so may represent a difference between producers and scroungers. Observing a producer switching to local search may be a good cue for scroungers to join them and replicate this search pattern. This may indicate an informational element to producer–scrounger dynamics, which has yet to receive extensive study. Furthermore, the decreasing efficiency of ARS with increasing group size may lead to fission of a group of foragers. Finally, interactions among foragers should affect the tendency to engage in local search or its duration. For example, interference competition among predators or increased vigilance of prey should impair ARS efficiency (Stillman, Goss‐Custard & Alexander, 2000; Scharf, Ovadia & Foitzik, 2012).

Evaluating spatial patterns of prey in parallel with documentation of ARS will be essential to understanding whether clumping of prey is a strict requirement for the latter. We suggest that methods quantifying the distance to nearest neighbour should be preferred because nearest‐neighbour distances are likely to be linked to the probability of encountering prey and switching to or remaining in local search. Where prey spatial pattern is unknown, ARS can be used to identify it and to understand better the scale at which specific animals perceive their habitat and forage. Furthermore, the presence of ARS can indicate that food is not easily perceived from a distance and could provide information on the forager's detection range. Finally, estimating hunger level is not trivial, and is often done indirectly, such as measuring the time since last feeding or the food required to reach satiation (e.g. Juell *et al*., 1994; Maselou, Perdikis & Fantinou, 2015). As the tendency to engage in local search and its duration are known to be affected by hunger, ARS may represent an indirect measure of hunger level. Finally, ARS models and experiments tend to focus on clumped sedentary prey and it remains an open question whether ARS is efficient when searching for moving prey (Ward & Webster, 2016, ch. 4). It is plausible that predators using ARS could capture moving prey efficiently as long as the prey remain clumped.

ARS is widespread across taxa, but its evolutionary history is unknown. It is recognized by a behavioural phenotype. We could be seeing convergent evolution of ARS in different taxa at the phenotypic level, or a mechanism conserved from relatively simple organisms, such as nematodes, to more complex ones. It will be intriguing to examine further whether the neurological mechanisms revealed in *C. elegans* are universal or similar in other animals. Furthermore, recent studies indicate the relevance of ARS to some aspects of human life, such as gathering food and memory search; it is possible that ARS may be applicable to other human behaviours (e.g. Hills *et al*., 2015; Mehlhorn *et al*., 2015; Schulz *et al*., 2019; Todd & Hills, 2020). This may be helpful, providing novel tools and ways of thinking from behavioural ecology to analyse phenomena in other disciplines faced with similar problems.

There is much more to discover about ARS. The increasing use of sophisticated tracking devices in behavioural and ecological studies opens new opportunities for researchers interested in ARS. We hope this review stimulates interest in this field and encourages researchers to examine this behaviour in non‐traditional ways and study systems.

## X. CONCLUSIONS

(1) Area‐restricted search (ARS) is a search pattern comprising the alternating use of extensive global search (exploration) and intensive local search (exploitation). The switch to local search is based on cues such as an encounter with food, odours, landmarks, or the memory/expectation of a target nearby. It is used by numerous animals from a wide variety of taxa.

(2) ARS is an efficient method to search for hidden targets where their general location, but not their exact location, is known. Methods applied to detect ARS should be adapted to the spatial scale relevant to the foraging behaviour of the focal animal. When possible, validation of episodes classified as ARS should be made using correlates of feeding behaviour. While ARS is mostly studied in a foraging context, it also can be used to search for single targets, such as nest sites or mates. Recent studies have applied ARS to describe psychological phenomena, such as memory search.

(3) When searching for multiple targets, such as prey items, ARS is most efficient when the targets are clumped in space. In such cases, it represents a fundamental mechanism of optimal foraging and patch departure decisions. The switch to local search, movement tortuosity, and its duration are all flexible. They depend, for example, on prey quality and density and the predator's hunger level. Several existing models suggest when ARS should be most efficient. Under some circumstances, such as a regular spatial distribution pattern of prey, ARS should fail.

(4) The neural mechanisms controlling ARS and ARS‐like behaviours have been investigated in *C. elegans*. Dopaminergic sensory neurons respond to prey capture by upregulating glutamatergic interneurons, which then activate motor neurons that increase turning and hence local search. ARS‐like behaviours in other species are modulated by similar mechanisms. Similar pathways may be involved in addiction patterns in humans and other animals.

(5) Future research could expand research on ARS to other fields and model ARS more broadly in the context of foraging, such as including the potential effects of competitors, predators, and ontogeny. Examining whether the same or similar neurological mechanisms explain ARS in unrelated animals will also be important to determine the extent to which it is phylogenetically conserved or a product of convergent evolution.
